# Impact of laparoscopic training, haptic feedback and visual haptic latency on virtual fine motor performance

**DOI:** 10.1038/s41598-025-18862-6

**Published:** 2025-09-12

**Authors:** Denise Tzolov, Jean-Paul Bereuter, Mark Enrik Geissler, Rona Berit Geissler, Grit Krause-Jüttler, Jürgen Weitz, Marius Distler, Florian Oehme, Annika Dix, Felix von Bechtolsheim

**Affiliations:** 1 Centre for Tactile Internet with Human-in-the-Loop (CeTI), TUD Dresden University of Technology, Dresden, Germany; 2 Department of Visceral, Thoracic and Vascular Surgery, Faculty of Medicine and University Hospital Carl Gustav Carus, TUD Dresden University of Technology, Fetscherstraße 74, 01307 Dresden, Germany; 3 Surgical Skills Lab Dresden, Medical Faculty and University Hospital Carl Gustav Carus, TUD Dresden University of Technology, Fetscherstraße 74, 01307 Dresden, Germany; 4 National Center for Tumor Diseases (NCT), NCT/UCC Dresden, a Partnership Between DKFZ, Faculty of Medicine and University Hospital Carl Gustav Carus, TUD Dresden University of Technology, and Helmholtz-Zentrum Dresden-Rossendorf (HZDR), Dresden, Germany

**Keywords:** Minimally invasive surgery, Haptic feedback, System latency, Medical research, Biomedical engineering

## Abstract

Advancements in minimally invasive surgery, including haptic feedback and telesurgery systems, offer potential benefits for surgical education and patient outcomes. However, the impact of general visuomotor skills (GVS), laparoscopic experience, and system latency on performance remains unclear. This study aimed to investigate how these factors interact during fine motor task execution. Fifty-seven medical students participated, with one subgroup (n = 29) receiving proficiency-based laparoscopic training and another (n = 28) without training. GVS were measured using the Purdue Pegboard Test, and laparoscopic skills via the Peg Transfer task. Participants then performed a Virtual Reality Wire Loop Game (WLG) under varying feedback conditions: visual feedback (real-time [VF0] or 200 ms delay [VF200]) and haptic feedback (real-time [HF0], delayed [HF200], or absent [noHF]). Performance metrics included error times, task completion times, and subjective difficulty ratings. Trained participants demonstrated significantly fewer errors, shorter completion times, and reduced instrument path length on the Peg Transfer task (all p < 0.01), whereas no between‐group differences emerged in Purdue Pegboard Test scores (all p > 0.05). A 200 ms visual delay significantly worsened WLG performance (p < 0.001) and increased perceived difficulty (p < 0.001). Real-time haptic feedback was rated more useful, particularly without visual delay, and its perceived benefit increased with LE (p < 0.001). Minimizing visual latency is essential in telesurgery. Real-time haptic feedback enhances performance, especially for experienced users.

**Supplementary Information:**

The online version contains supplementary material available at 10.1038/s41598-025-18862-6.

## Introduction

Minimally invasive procedures, especially robotic surgery, have revolutionized surgery by making it possible to perform complex procedures with high precision. For patients in particular, this has resulted in less wound trauma and therefore less pain and faster recovery^1^. Despite these advancements, a significant limitation remains: the lack or deterioration of tactile feedback. Traditional surgical techniques allow surgeons to rely on their sense of touch to assess tissue properties and make nuanced adjustments during a procedure. This sensory feedback is crucial for tasks such as determining tissue texture, tension, and consistency. The importance of tactile input has been emphasized in surgical performance studies and meta-analyses evaluating haptic feedback systems^2^, and training-based assessments have also noted the challenges posed by limited sensory cues during laparoscopic tasks—especially during more complex or force‑dependent tasks^3^.

To address this limitation, haptic feedback systems have been integrated into minimally invasive and robotic surgery platforms. These technologies aim to restore tactile sensations via the instruments used, enabling surgeons to better perceive and modulate their interactions with tissue. Integrating haptic technology allows these systems to simulate the feel of real tissues, offering a more intuitive and responsive surgical experience^4^. This advancement enhances the surgeon’s ability to perform delicate maneuvers, improves force control^5–8^, reduces the learning curve for new surgeons^9^, and ultimately contributes to better surgical outcomes^10^.

In parallel, simulation environments and virtual reality (VR) platforms have emerged as valuable tools for surgical training and research. These systems provide controlled, repeatable conditions under which core sensorimotor processes can be investigated. General visuomotor skills (GVS)—such as visual-spatial ability, hand–eye coordination, dexterity, and bimanual coordination—are essential for the successful execution of minimally invasive procedures^11^. GVS are critical for coordinating physical movements into accurate and efficient action sequences, particularly when direct vision and tactile cues are limited. Sensorimotor expertise not only manifests in more efficient movement execution but also in an improved ability to perceive and process incoming sensory information^12^, underscoring its importance in the operating room.

To examine these sensorimotor processes in a controlled and reproducible environment, simplified virtual tasks such as the Wire Loop Game (WLG) have been adopted in surgical research. The WLG requires users to guide a virtual object along a constrained path with high precision, penalizing contact errors and timing performance—core elements of laparoscopic skill. Prior studies have demonstrated that performance in tasks like the WLG correlates with laparoscopic proficiency, supporting its use as a proxy for real-world surgical dexterity and fine motor control. However, beyond visuomotor coordination, other factors also influence performance in these environments. One such factor is system latency—the delay between user input and system response^13^. From a neuroscience perspective, even brief feedback delays can destabilize movement, particularly in skilled tasks that depend on predictive motor control^14^. To maintain accuracy during action, the brain generates internal forward models that predict sensory consequences of motor commands. These predictions are compared with actual feedback, allowing for rapid error correction^15–17^. When discrepancies—termed sensory prediction errors—are too large or feedback is too delayed, motor adaptation may fail, leading to performance impairments^18^. Research in sensorimotor neuroscience suggests that delays greater than 100 ms can significantly impair timing and accuracy in fine motor tasks.

These insights have direct implications for telesurgical applications^19,20^. In remote surgery systems, communication delays as small as 100–200 ms have been shown to degrade precision and increase cognitive load. For instance, Xu et al. demonstrated that surgical performance deteriorates exponentially with increasing latency in a Da Vinci simulator environment^21^. Moreover, Khan et al. reported that delayed visual feedback negatively impacts fine motor surgical tasks in augmented reality systems^22^. These applied findings underscore the importance of minimizing system latency to ensure real-time, high-precision control in surgical environments.

Laparoscopic experience (LE) further modulates these dynamics. Surgeons with more training typically perform better in robotic and minimally invasive procedures due to improved motor strategies and error correction mechanisms^23,24^. Experience also shapes how sensory feedback is used and interpreted during task performance. Despite this, the interactions between GVS, LE, haptic feedback, and system latency remain poorly understood.

This study investigated how delayed visual and haptic feedback influence fine motor performance in a VR Wire Loop Game (WLG), with a particular focus on the roles of laparoscopic experience (LE) and general visuomotor skills (GVS). The WLG has been widely used in surgical research as a simplified, controlled task that captures key demands of laparoscopic performance, including bimanual coordination, movement precision, and error avoidance. Prior studies have linked WLG-like tasks to real-world laparoscopic proficiency, supporting their use as valid proxies for assessing fine motor control in a virtual setting. We hypothesized that delayed visual feedback would impair performance, reflected in longer error times and trial durations, regardless of experience (H1). We further expected that the absence or delay of haptic feedback would reduce performance compared to real-time feedback conditions (H2, H3). Lastly, we anticipated that participants with higher levels of LE and GVS would generally perform better across conditions (H4). An exploratory analysis additionally examined how these variables interact to influence subjective perceptions of task difficulty and the usefulness of haptic feedback. A better understanding of these interdependencies may guide the development of optimized feedback systems and training protocols for minimally invasive and robotic-assisted surgery, particularly in settings involving latency, such as telesurgery.

## Materials and methods

This study was conducted in accordance with the Declaration of Helsinki^25^. In addition, the study was approved by the ethics committee of the Technische Universität Dresden in the context of a larger research project (SR-EK-207052020; date of approval: 07.07.2020; date of approval for amendment regarding the usage of the VR environment: 24.08.2022), and written informed consent was obtained from all subjects. Of note, each participant had the opportunity to withdraw participation at any time during the experiment. After testing, participants received 10 Euros as gratification for their support in this study.

### Participants

This study was performed at the University Hospital Dresden between April and June 2023. The study cohort consisted of 57 medical students and contained two subgroups: One subgroup did previously participate in a laparoscopic training course (n = 29) and has laparoscopic experience while the other subgroup did not (n = 28) and therefore has no laparoscopic experience. All participants of the first mentioned subgroup (n = 29) that did previously participate in a laparoscopic training course were trained according to a modified *Fundamentals of Laparoscopic Surgery* (FLS) training curriculum until they achieved a predefined threshold level of proficiency, which was described in detail previously^26^. In our study, participants were considered to have reached the predefined proficiency level when they completed the task in under 120 s without committing any errors.

### Task and procedures

First, the participants’ laparoscopic skill level and their GVS were evaluated to later investigate how the laparoscopic experience and fine motor skills influence performance (see Supplementary Fig. 1). The laparoscopic skill level was measured using the ForceSense device (Medishield B.V., Delft, The Netherlands) within a laparoscopic box trainer. The device used was able to directly measure the task completion time as well as the distance that each instrument tip travels during the task (= path length). The participants’ laparoscopic experience was objectively evaluated based on their error rate, their task completion time and their total path length for the Peg transfer task. The error rate was determined by the cumulated number of Pegs dropped to the floor. The GVS were assessed with the Purdue Pegboard Test (PPT). Specifically, we followed the instructions of the official test manual of the Purdue Pegboard Test Model 32020A as written by Lafayette Instruments. Before the test, the pegboard was prepared according to the handedness of the participant. Then, the subjects were instructed that the PPT is a test to see how quickly and accurately they can work with their hands and that the test consists of 4 subtasks. Before beginning each battery of the test, they would be told what to do and then they had an opportunity to practice. Each participant performed each subtask once. For the first three tasks (right hand, left hand, both hands), they were given a time limit of 30 s. For the last task (assembly), participants were given one minute to assemble as many components as possible.

**Fig. 1:**
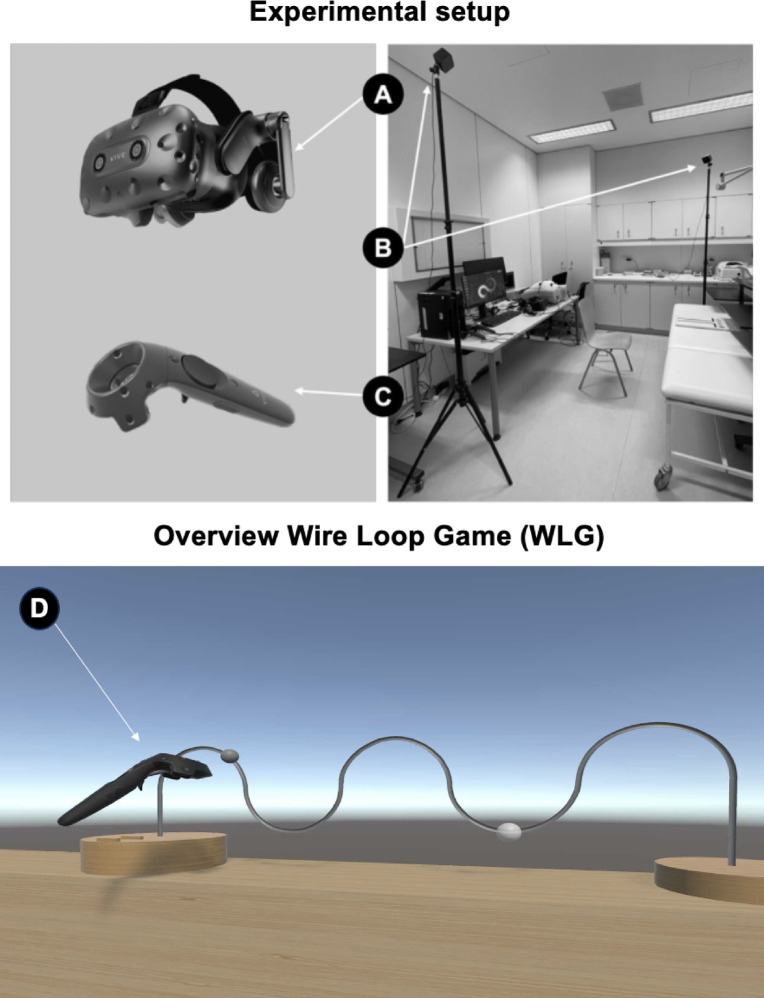
Overview of the experimental setup and the conducted Virtual Reality Wire Loop Game (WLG). For the completion of the task the HTC VIVE Pro Virtual Reality Kit (HTC Corporation, Taoyuan, Taiwan) was used in combination with the Virtual Reality Software SteamVR (Valve Corporation, Bellevue, WA). (**A**) HTC VIVE Pro Headset. (**B**) Basis stations in the experimental room. (**C**) Controller of the HTC VIVE Pro Virtual Reality Kit. (**D**) Overview of the conducted VR Wire Loop Game.

Afterwards, the main task of the study was conducted. This task employed the Virtual Reality (VR) version of a classic fine motor skill task, also known as Wire Loop Game (WLG). As shown in Fig. 1A–C, the VR game was displayed using the HTC VIVE Pro Virtual Reality Kit (HTC Corporation, Taoyuan, Taiwan) (HTC VIVE Pro) in combination with the Virtual Reality Software SteamVR (Valve Corporation, Bellevue, WA). The experiment was conducted using an HTC Vive Pro headset (90 Hz refresh rate), SteamVR 2.0 tracking with lighthouse base stations (60 Hz), and controllers with an internal update rate of 360 Hz. The Unity engine (version 2021.1.15f1) was used in combination with the SteamVR plugin. Based on prior studies, the motion-to-photon (MTP) latency of the HTC Vive Pro system is estimated to be approximately 31 ms for sudden movements and up to 47.9 ms when accounting for Unity engine-induced delays^27–29^. The hand-controller latency has been reported to be approximately 4 ms. Together, these findings suggest a baseline end-to-end system latency of approximately 60 ms in our setup. The conducted VR WLG required guiding a ball along a bent wire without losing contact to the wire (see Fig. 1D). When leaving the wire with the ball, the participants received haptic feedback (vibration of controller) in some conditions, which only disappeared again when contact with the wire was successfully established. Additionally, visual and/or haptic feedback was 200 ms delayed in some trials.

We selected a visual delay of 200 ms based on previous empirical findings indicating that this value represents a critical threshold at which performance decrements begin to emerge in tasks involving fine motor control. For instance, Dix et al. observed significant impairments in task duration and error time during a complex motor task at delays of 200 and 300 ms^30^. Similarly, in medical contexts, Xu et al. and Perez et al. reported measurable performance declines beginning at 200 to 300 ms, with more severe deficits at higher latencies^21,31^. Moreover, Kumcu et al. and Lum et al. identified performance impairments at latencies as low as 160 to 250 ms, underscoring the variability of sensitivity depending on task type and setting^32,33^. Importantly, Khan et al. highlighted 200 ms as the threshold in an augmented reality context, beyond which motor performance significantly deteriorated^22^. These findings collectively justify the selection of 200 ms as a conservative yet meaningful delay value, particularly for studying subtle performance changes without inducing ceiling effects. Consequently, all participants performed the WLG under the combination of the following visual and haptic feedback conditions:

- *Visual Feedback (VF)*: Real-time (VF0) vs. delayed (VF200).
- *Haptic Feedback (HF)*: Real-time (HF0) vs. delayed (HF200) vs. no feedback (noHF).

In conditions labeled “VF200” an additional 200 ms delay was artificially imposed on top of this baseline latency, resulting in a total signal latency of approximately 260 ms from user input to visual or haptic feedback. These six feedback combinations were presented to the participants a total of four times in a randomised order:

- VF0-HF0: Real-time visual and haptic feedback.
- VF0-HF-200: Real-time visual feedback and 200 ms delayed haptic feedback.
- VF0-noHF: Real-time visual feedback and no haptic feedback.
- VF200-HF0: 200 ms delayed visual feedback and real-time haptic feedback.
- VF200-HF200: 200 ms delayed visual and haptic feedback.
- VF200-noHF: 200 ms delayed visual feedback and no haptic feedback.

The six experimental conditions of the WLG were presented to each participant four times in a pseudorandomized order, resulting in 24 trials. Randomization was performed individually for each participant within four blocks, with each block containing all six conditions in a randomized sequence. This block-wise pseudorandomization was used instead of full randomization across all trials to ensure early exposure to all feedback conditions and to reduce the likelihood of repeated consecutive presentations of the same condition, thereby minimizing potential learning or order effects. No counterbalancing across participants was implemented. During the experiment, the Virtual Reality Sickness Questionnaire (VRSQ) was utilised to investigate if the participants were negatively affected by the virtual reality experience^34^. Each testing lasted about 30–40 min.

### Endpoints

The impact of the described feedback modalities on the VR WLG was assessed using different objective metrics such as the error time and the task completion time. While the task completion time was defined as the time needed to conduct the complete task per trial, the error time was defined as the time incorrectly moving the ball outside the wire per trial. In addition, subjective metrics were collected after each trial by asking the participants to rate the perceived task difficulty and usefulness of the haptic feedback based on a 6-point Likert scale ranging from 1 (“very difficult”/“not useful”) to 6 “not difficult”/“very useful”).

### Data analysis

For data processing and statistical analyses, SPSS version 28 (IBM Corp., Armonk, NY, USA) was used. Outliers were excluded. Specifically, 23 trials were excluded based on within-subject outlier detection, defined as values ± 3.29 SD from a participant’s mean for error duration or speed. After aggregation (i.e., computing the mean per condition for each participant), a second outlier analysis was conducted across the full sample using the same criterion, leading to the exclusion of three participants. To calculate a variable for GVS, the sum of the individual tasks of the PPT was determined and the sum of the subscores was then z-standardised. In our analysis, the three variables—Peg Transfer error count, completion time, and path length—were z-standardized to ensure comparability despite differing scales. Each was then inverted so that higher values uniformly reflected better performance. This inversion simplifies interpretation of the covariate, aligning it conceptually with GVS, where higher values also represent superior performance. We selected these three metrics because they closely match the performance indicators assessed in the VR task (i.e. errors, time, and distance from the ghost ball). While not all of these were included in the final outcome analysis of the VR experiment, they reflect the core behavioral dimensions the task was designed to measure. We applied equal weighting to the three z-standardized and inverted components because we considered all three equally relevant indicators of performance within the context of the task. The effects on performance and subjective metrics were analysed using repeated measures analyses of variance with covariates (RM-ANCOVAs). Visual feedback (real-time, delayed) and haptic feedback (real-time, delayed, no HF) served as within-subject factors and the LE and GVS as covariates to examine their effects on the dependent variables. The dependent variables were error time, task completion time, perceived task difficulty and perceived usefulness of the haptic feedback. Further simple repeated measures analyses of variance (ANOVAs) with VF and HF as within-subject factors were computed for those effects that were independent of the covariates. F-tests were used for both RM-ANCOVAs and ANOVAs. Significant results were reported with p-values and the effect size partial eta squared (ηp^2^). According to Richardson, a ηp^2^-value of 0.01 indicates a small effect, 0.06 a medium effect and 0.14 a large effect^35^. To capture individual differences in skill more precisely, laparoscopic experience (LE) and general visuomotor skills (GVS) were treated as continuous covariates rather than categorical grouping factors. This approach avoids arbitrary dichotomization and increases the sensitivity of the statistical models by accounting for within-group variance. Including both LE and GVS as covariates allowed us to disentangle the distinct contributions of laparoscopic training and general motor coordination to task performance, providing a more nuanced understanding of the factors that influence fine motor control under varying sensory feedback conditions. The objective variables error time and task completion time have already been investigated in previous research and therefore served as core analysis in this study^30^. A p-value smaller than 0.025 (after Bonferroni correction for multiple testing) was considered statistically significant exclusively to the two primary dependent variables: error time and task completion time. All analyses involving subjective ratings were considered exploratory and thus interpreted using the conventional significance threshold of 0.05.

## Results

### Basic participant characteristics

**Fig. 2:**
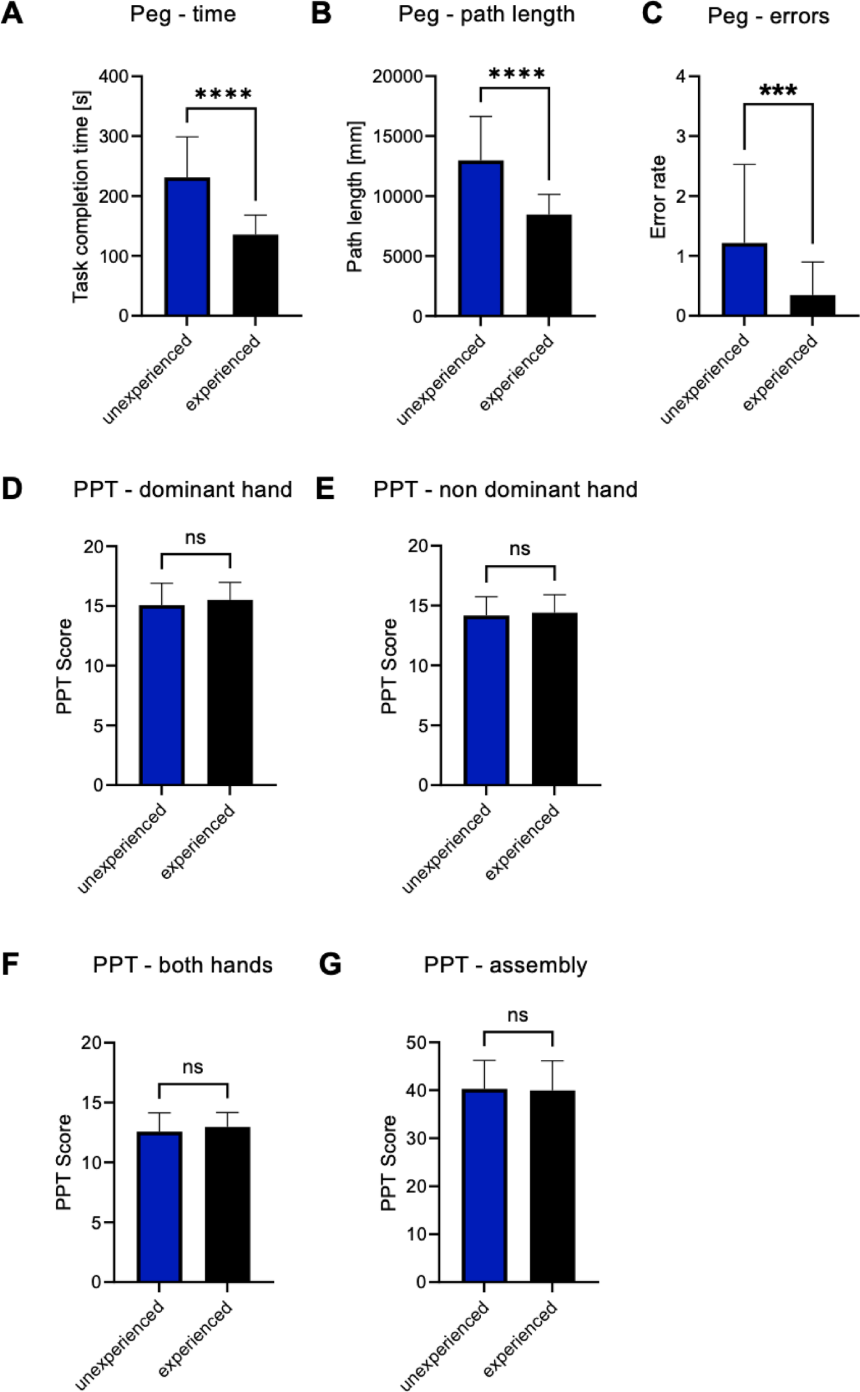
Evaluation of the participants’ laparoscopic skill level on the basis of their performance in the Peg transfer task and evaluation of their GVS, as indicated by their performance in the PPT. (**A**) Comparison of the task completion times between the groups of inexperienced and experienced medical students in the Peg transfer task. (**B**) Comparison of the total path length between the groups of inexperienced and experienced medical students in the Peg transfer task. (**C**) Comparison of the error rates between the groups of inexperienced and experienced medical students in the Peg transfer task. (**D**) Comparison of the results of the dominant hand between the group of inexperienced and experienced medical students in the Purdue Pegboard Test (PPT). (**E**) Comparison of the results of the nondominant hand between the group of inexperienced and experienced medical students in the PPT. (**F**) Comparison of the results of both hands between the group of inexperienced and experienced medical students in the PPT. (**G**) Comparison of the results of the assembly between the group of inexperienced and experienced medical students in the PPT. *p < 0.05, **p < 0.01, ***p < 0.001, ****p < 0.0001.

Fifty-seven medical students participated in this trial. The participants had a median age of 23.7 years (SD = 2.1 years). All participants were students who had completed at least the second year of their medical studies. The study group comprised 26 male and 31 female participants. Most students were right-handed (94.7%) and the minority left-handed (5.3%). The study cohort consisted of two subgroups. One subgroup did not previously participate in a FLS based laparoscopic training curriculum (n = 28) while the other subgroup did so (n = 29). Both subgroups were similar concerning age (inexperienced: 23.2 years ± 1.8 years vs. experienced: 24.2 ± 2.2 years; p = 0.06) and the dominant hand (inexperienced: 96.4% right-handed vs. experienced: 93.1% right-handed; p = 57) while the sex distribution differed between both subgroups (inexperienced: 28.6% males vs. experienced: 62.1% males, p = 0.01) (see Table 1). Based on the different experience levels, the participants of the experienced subgroup showed significantly less major surgical errors (inexperienced: 1.2 Peg drops vs. experienced: 0.34 Peg drops; p < 0.01), faster task completion times (inexperienced: 231.3 s ± 67.5 s vs. experienced: 135.4 s ± 32.9; p < 0.01) and reduced path lengths (inexperienced: 12,977.05 mm ± 3,658.62 mm vs. experienced: 8,457.67 mm ± 1,689.62; p < 0.01) (see Fig. 2). Interestingly, no significant differences were seen between both groups in the PPT (see Table 2). Regarding prior experience with virtual reality (VR), participants’ gaming experience was assessed. Approximately 5.3% of the sample reported currently playing video games, while an additional 19.3% had past gaming experience (see Supplementary Table 1). Weekly gaming time was recorded and analyzed in relation to fine motor performance. No evidence was found that prior VR exposure systematically affected the results.

**Table 1:** Basic participant characteristics.

Items	Inexperienced (n = 28)	Experienced (n = 29)	p value
Age, mean [mean (SD)]	23.2 (1.8)	24.2 (2.2)	0.06
Sex, number [n (%)]			0.01
Male	8 (28.6)	18 (62.1)	
Female	20 (71.4)	11 (37.9)	
Dominant hand [n (%)]			0.57
Right hand	27 (93.4)	27 (93.1)	
Left hand	1 (3.6)	2 (6.9)	

Comparison of basic participant characteristics between the groups of inexperienced and experienced medical students

**Table 2:** Evaluation of laparoscopic skill and GVS performance (PPT), comparing inexperienced and experienced students.

Items	Inexperienced (n = 28) mean (SD)	Experienced (n = 29) mean (SD)	p value
Purdue Pegboard Test			
Right hand	15.1 (1.8)	15.5 (1.5)	0.12
Left hand	14.2 (1.6)	14.4 (1.5)	0.60
Both hands	12.6 (1.6)	12.97 (1.2)	0.22
Assembly	40.3 (5.9)	40 (6.2)	0.95
Peg transfer			
Errors	1.2 (1.3)	0.3 (0.6)	** < 0.01**
Task completion time	231.3 (67.5)	135.4 (33.0)	** < 0.01**
Total path length	12,977.1 (3,658.6)	8,457.7 (1689.6)	** < 0.01**

Comparison between the group of inexperienced and experienced medical students in terms of basic participant characteristics and their performance in GVS tasks such as the PPT and the Peg transfer task. Groups were compared using an unpaired Student’s t-test. Significant p values are highlighted in bold.

### Impact of LE on VR task performance

The analyses did not reveal any significant effects of LE on error time or trial duration in the conducted VR WLG. In the 2 × 3 ANCOVA for error time, neither the main effect of LE (F(1, 54) = 0.750, p = 0.390) nor any interactions involving LE reached significance (VFxLE: F(1, 54) = 0.614, p = 0.437; HFxLE: F(2, 108) = 2.060, p = 0.132; VFxHFxLE: F(2, 108) = 0.949, p = 0.390). Similarly, in the 2 × 3 ANCOVA for trial duration, LE showed no significant main effect (F(1, 54) = 0.003, p = 0.954) and did not interact significantly with visual or haptic feedback significance (VFxLE: F(1, 54) = 0.133, p = 0.716; HFxLE: F(2, 108) = 0.051, p = 0.950; VFxHFxLE: F(2, 108) = 0.023, p = 0.978). These results show that LE did not influence VR task performance. Instead, error time and duration were primarily affected by sensory feedback conditions and GVS. Given the absence of significant effects, LE was excluded from further analyses of the primary dependent variables error time and task completion time (i.e., the 2 × 3 ANOVAs).

### Impact of visual and haptic feedback latencies on VR task performance

In the conducted VR WLG, a visual feedback latency of 200 ms had a large effect on both objective performance measures (error time: F(1, 56) = 627.77, p < 0.001, ηp^2^ = 0.92; task completion time: F(1, 56) = 80.52, p < 0.001, ηp^2^ = 0.59). Specifically, post-hoc tests with Bonferroni correction showed that delayed visual feedback substantially increased both error time (*p* < 0.001, *MDiff* = 2832.78 ms, 95%-CI [2606.288, 3059.264], VF0: *M* = 713.502, *SE* = 58.678; VF200: *M* = 3546.278, *SE* = 113.147), and task completion time (*p* < 0.001, *MDiff* = 2.646 s, 95%-CI [2.055, 3.236], VF0: *M* = 6.224, *SE* = 0.059; VF200: *M* = 8.870, *SE* = 0.339). In contrast to visual feedback delays, the integration and delay of haptic feedback did not significantly impact performance (error time: *F*(2, 112) = 0.573, *p* = 0.566; HF0: *M* = 2108.599, *SE* = 81.289; HF200: *M* = 2114.413, *SE* = 77.300; noHF: *M* = 2166.659, *SE* = 76.251; task completion time: *F*(2, 112) = 0.069, *p* = 0.933; HF0: *M* = 7.557, *SE* = 0.196; HF200: *M* = 7.528, *SE* = 0.183; noHF: *M* = 7.556, *SE* = 0.216). However, the influence of haptic feedback became relevant when considering participants’ GVS.

### Interaction between VF, HF and GVS on VR task performance

**Fig. 3:**
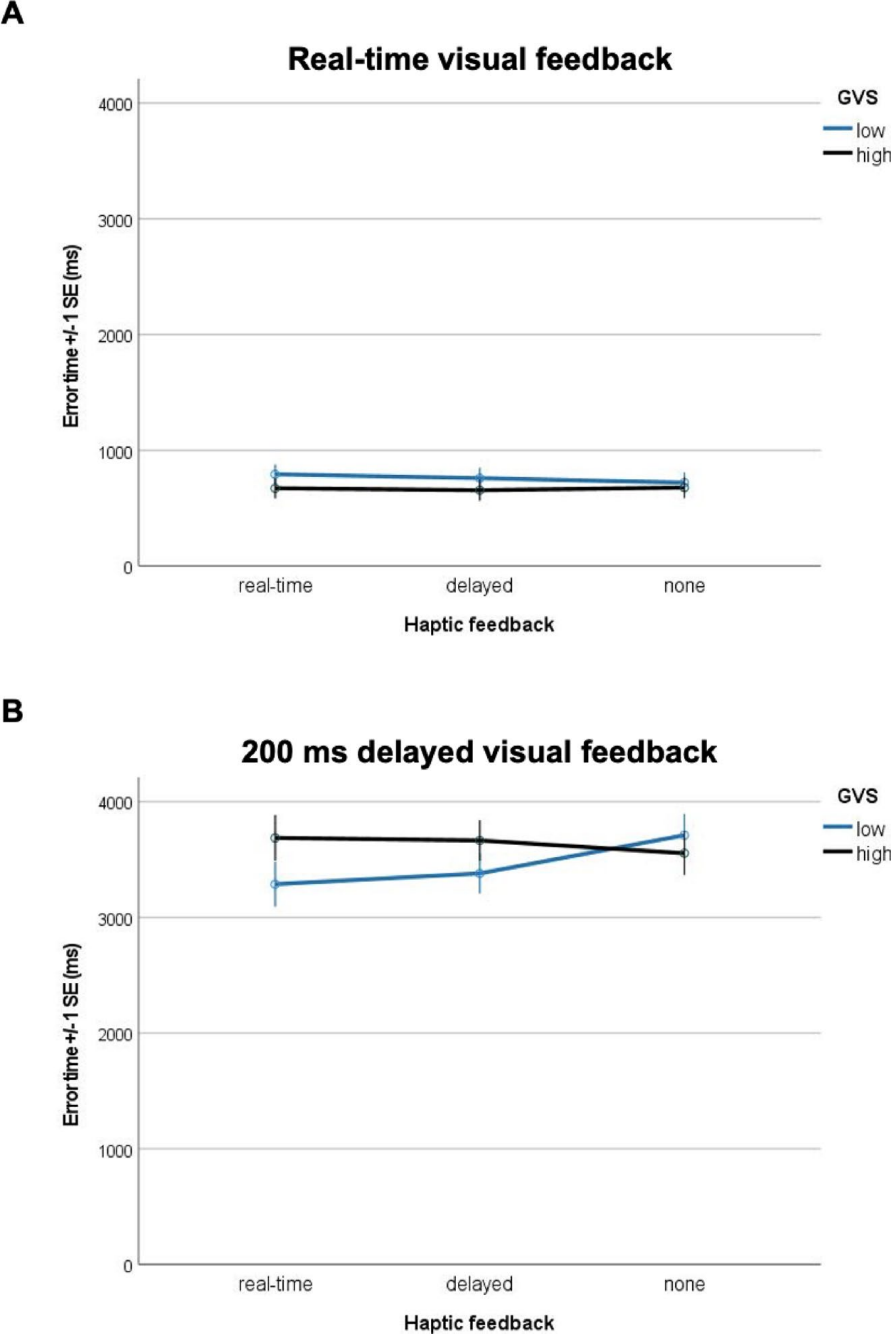
Effect of varying visual and haptic feedback latencies on the participants’ error times (ms) based on z-standardised GVS. (**A**) Impact of real-time visual feedback in combination with varying haptic feedback options based on z-standardised GVS scores. A median split was conducted to categorize participants into “low GVS” and “high GVS”. (**B**) Impact of 200 ms delayed visual feedback in combination with varying haptic feedback options based on z-standardised GVS scores. A median split was conducted to categorize participants into “low GVS” and “high GVS”.

**Fig. 4:**
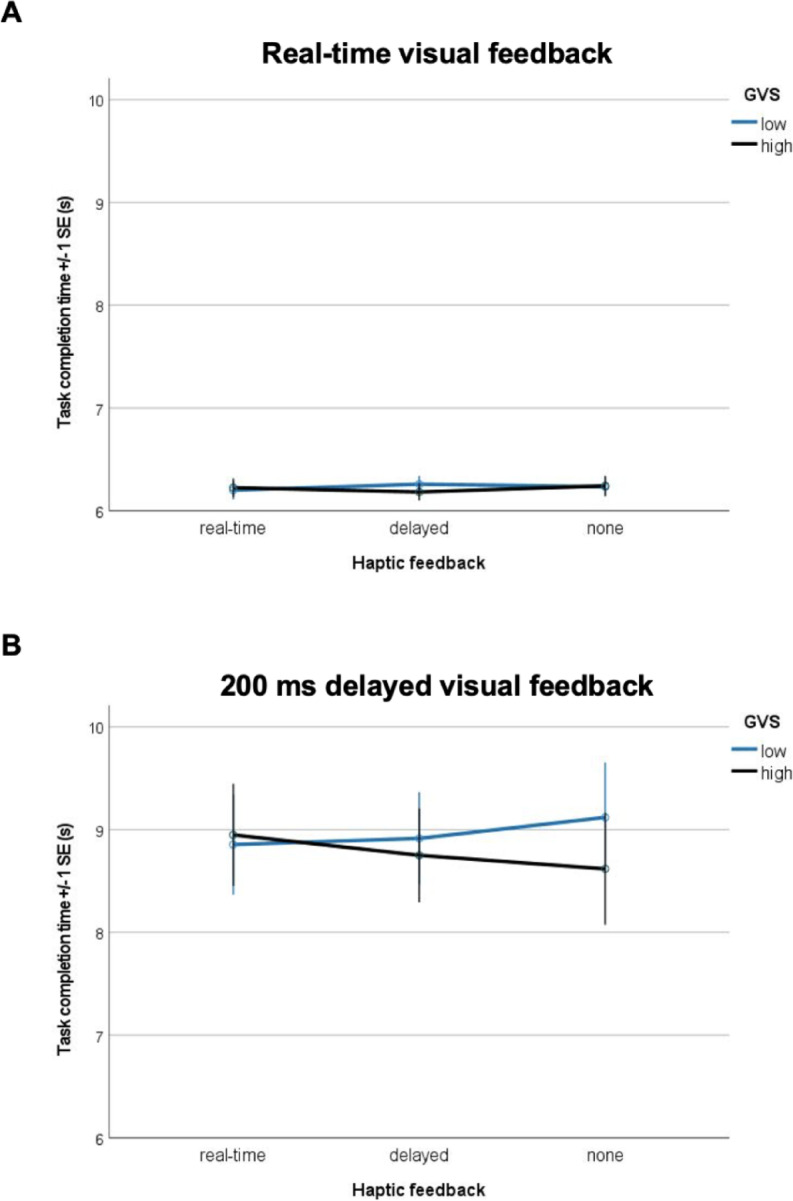
Effect of varying visual and haptic feedback latencies on the participants’ task completion times (s) based on z-standardised GVS. (**A**) Impact of real-time visual feedback in combination with varying haptic feedback options based on z-standardised GVS scores. A median split was conducted to categorize participants into “low GVS” and “high GVS”. (**B**) Impact of 200 ms delayed visual feedback in combination with varying haptic feedback options based on z-standardised GVS scores. A median split was conducted to categorize participants into “low GVS” and “high GVS”.

A significant three-way interaction between visual feedback (VF), haptic feedback (HF), and general visuomotor skill (GVS) was found for both error time (F(2, 108) = 5.478, p = 0.005, ηp^2^ = 0.09, , see Fig. 3) and task completion time (F(2, 108) = 3.959, p = 0.022, ηp^2^ = 0.07, see Fig. 4), indicating a medium-sized effect of GVS depending on the VF and HF condition. Under real-time visual feedback (VF0), GVS had no significant effect on error time or task duration, regardless of the haptic feedback condition (all p > 0.05). Under delayed visual feedback (VF200), GVS effects varied by haptic feedback condition: Under conditions of delayed visual feedback (VF200), the effect of general visuomotor skill (GVS) on task performance varied depending on the type of haptic feedback. When no haptic feedback was provided (no-HF), higher GVS was associated with significantly fewer errors (t = − 3.348, p = 0.001, ηp^2^ = 0.17) and shorter task completion times (t = − 3.174, p = 0.002, ηp^2^ = 0.16). In contrast, when real-time haptic feedback was present (HF0), this relationship reversed: higher GVS predicted increased error time (t = 3.045, p = 0.003, ηp^2^ = 0.14) and longer task duration (t = 2.997, p = 0.004, ηp^2^ = 0.14). No significant GVS effects were observed when both visual and haptic feedback were delayed (HF200; all p > 0.05). Thus, when vision was delayed, GVS helped only when haptic feedback was absent. Conversely, in the presence of real-time haptic feedback under visual delay, higher GVS paradoxically predicted worse performance. This suggests that high-GVS individuals may be more vulnerable to sensory conflict when feedback modalities are temporally misaligned.

### Subjective perceptions of task difficulty and usefulness of HF

Regarding perceived task difficulty, main effects with large effect sizes for both VF (*F*(1, 56) = 757.491, *p* < 0.001, ηp^2^ = 0.93) and HF (*F*(2, 112) = 8.961, *p* < 0.001, ηp^2^ = 0.14) could be found. Post-hoc tests with Bonferroni correction showed that tasks performed without visual delays were perceived as easier (p < 0.001, MDiff = 2.212, 95%-CI [2.051, 2.374]). Moreover, without haptic feedback the task was perceived as easier compared to the conditions with haptic feedback (real-time HF: p = 0.014, MDiff = -0.133, 95%-CI [− 0.245, − 0.021]; delayed HF: p < 0.001, MDiff = − 0.180, 95%-CI [− 0.293, − 0.067]; VF0-HF0: *M* = 2.860, *SD* = 0.656; VF0-HF200: *M* = 2.906, *SD* = 0.773; VF0-noHF: *M* = 2.654, *SD* = 0.745; VF200-HF0: *M* = 5.023, *SD* = 0.526; VF200-HF200: *M* = 5.070, *SD* = 0.600; VF200-noHF: *M* = 4.964, *SD* = 0.626). Taking into account the participants’ experience levels, medium effects for both covariates LE and GVS became visible. The significant main effect of LE (*F*(1, 54) = 4.085, *p* = 0.048, ηp^2^ = 0.07) reveals that with increasing LE, the task was perceived as easier. Additionally, there was a significant threeway-interaction-effect of VF, HF and GVS (*F*(2, 108) = 4.365, *p* = 0.015, ηp^2^ = 0.08), showing a differing impact of GVS particularly between the two conditions VF0-noHF and VF0-HF0. When there was real-time visual feedback without haptic feedback, participants with higher GVS rated the task as more difficult compared to those with lower GVS. However, when there was real-time visual feedback and real-time haptic feedback, the task was rated as easier with increasing GVS (*t* = -3.276, *p* = 0.002, ηp^2^ = 0.16).

Regarding the perceived usefulness of haptic feedback, there were significant effects for visual feedback (*F*(1, 54) = 66.441, *p* < 0.001, ηp^2^ = 0.55) and the interaction between VF and LE (*F*(1, 54) = 6.246, *p* = 0.016, ηp^2^ = 0.10). Real-time haptic feedback was generally perceived as more beneficial in tasks without visual delays (*p* < 0.001, *MDiff* = 0.802, 95%-CI [0.605, 0.999]; VF0-HF0: *M* = 4.618, *SD* = 0.697; VF0-HF200: *M* = 4.589, *SD* = 0.738; VF200-HF0: *M* = 3.752, *SD* = 0.958; VF200-HF200: *M* = 3.852, *SD* = 1.007). Further, in these tasks, the perceived usefulness of haptic feedback increased significantly with the level of LE. However, if visual feedback was delayed, haptic feedback was rated as less helpful with increasing LE (*t* = 2.285, *p* = 0.026, ηp^2^ = 0.09), highlighting its subjective value in skilled users depending on visual feedback.

## Discussion

This study investigated the influence of LE, GVS, VF, HF and system latency on fine motor task performance, using a VR WLG. We found that a 200 ms visual delay significantly increased error time by 2.8 s (ηp^2^ = 0.92), while a 200 ms haptic delay had no significant effect on performance. Notably, GVS interacted with the feedback conditions: under delayed vision, individuals with higher GVS performed better when haptic feedback was absent, but worse when real-time haptic feedback was present.

A major contribution of this study lies in its combined factorial manipulation of visual and haptic delays—a design that extends beyond prior studies, which typically tested only one feedback modality in isolation. By examining VF (VF0 vs. VF200) and HF (HF0, HF200, or HF0) in a single framework while controlling for both GVS and LE as continuous covariates, we revealed context-dependent effects of expertise and cross-modal feedback interactions. Individuals with high GVS performed best under VF200 + no-HF, but their performance deteriorated under VF200 + HF0. This challenges the assumption that sensorimotor expertise universally protects against degraded feedback, and instead suggests that expertise can become maladaptive under conflicting sensory conditions.

At first glance, the finding that high-GVS participants performed worse under delayed visual feedback combined with real-time haptic feedback appears counterintuitive, given that visuomotor expertise is typically thought to buffer against sensory degradation^36,37^. However, this result aligns with theories of experience-dependent sensory integration. Experts often develop refined internal models that rely heavily on stable visual input. When visual information becomes delayed while haptic feedback remains synchronous, the resulting asynchronous sensory streams can create a mismatch that disrupts these internal models. In contrast, individuals with lower GVS may use more flexible, feedback-driven strategies, making them less susceptible to such conflict.

This interpretation is consistent with multisensory integration theory, particularly the notion of statistically optimal integration, which proposes that the brain weights each sensory modality based on its perceived reliability^38^. High-GVS individuals may downweight delayed visual input and rely more on proprioception or motor predictions. However, when immediate haptic input contradicts delayed visual feedback, this conflict may result in degraded performance. This mechanism mirrors findings from other domains: Thalassinos et al. showed that expert athletes became more vulnerable to multisensory disruptions, while Kulpa and Pfordresher reported that expert musicians were more impaired by audiovisual asynchronies than novices^39,40^. These effects may reflect a form of “negative transfer”, where highly optimized sensorimotor strategies become maladaptive under altered sensory conditions. Our findings offer preliminary support for this phenomenon in haptic-visual contexts.

Visual delay alone emerged as a powerful disruptor of performance, echoing prior research in telesurgical and VR domains showing that delays above 100 ms degrade accuracy and timing exponentially^21,22^. In contrast, haptic delays up to 200 ms were tolerated, consistent with work by Jay and Hubbold 2005 or Gourishetti et al.^41,42^ who found minimal impact of similar delays in simple tasks. Our findings suggest that while visual latency undermines forward model predictions, modest haptic delay may be less disruptive in tasks like the WLG, which involve relatively predictable and low-force interactions.

The subjective data further reinforce the experience-dependent nature of sensory integration. While novices perceived real-time haptic feedback as more helpful under visual delay, experienced participants perceived it as less useful. This divergence suggests that experts dynamically reweight sensory inputs depending on task demands and feedback reliability. When visual input is compromised, they may rely more on internal models and suppress misaligned feedback. This aligns with predictive coding accounts and research on sensory suppression and attentional control in experts^12,43,44^. Novices, lacking robust internal models, may instead benefit from any available external cues, regardless of synchrony.

Interestingly, participants rated tasks without haptic feedback as subjectively easier, despite haptic feedback being intended to aid performance. This may reflect a lack of prior experience with haptic systems, particularly among novices. Vibration-based cues, when unexpected or ambiguous, may be perceived as disruptive error signals rather than informative guidance—especially in the absence of clear task associations. Prior research shows that unfamiliar multisensory inputs can increase cognitive load or attentional demands if their meaning is unclear or inconsistent with other modalities^45,46^.

From a technical perspective, our study utilized the HTC VIVE Pro with SteamVR, offering improved spatial and temporal precision compared to earlier systems. This, combined with our use of continuous covariates (LE and GVS), enabled more sensitive detection of performance variability and expert-novice differences than traditional between-group designs. To our knowledge, this is one of the first studies to simultaneously manipulate both visual and haptic latency, assess their interaction with expertise, and interpret the findings within a sensory reweighting framework.

### Limitations

While this study offers valuable insights, it has certain limitations. First, the cohort was composed entirely of medical students, which may limit the generalizability of the findings to more experienced clinical populations. Medical students, regardless of whether they have completed simulation-based training or not, are still at an early stage of their surgical education, and their motor strategies, cognitive load management, and adaptive responses likely differ substantially from those of residents or board-certified surgeons. As such, the observed effects of haptic and visual feedback, as well as vestibular perturbation, may not reflect how these factors interact in real-world surgical practice. Future research should aim to include surgical residents and attending surgeons to determine whether the same patterns of GVS-HF-VF interactions hold in more experienced users.

Second, the operationalization of LE was based solely on participants’ completion of a single FLS-based proficiency benchmark (= Peg transfer task), which introduces an artificial dichotomy between “experienced” and “novice” participants. This definition does not reflect the depth and breadth of real-world surgical experience, where procedural exposure can number in the hundreds or thousands. For instance, an attending surgeon who has performed 500 laparoscopic cholecystectomies is likely to exhibit different sensorimotor adaptations, error correction strategies, and decision-making processes than a medical student who has passed a simulation module. Therefore, findings related to LE in this study must be interpreted with caution, and should not be generalized to represent the behavior of clinically experienced surgeons.

Third, while the VR WLG task serves as a valuable proxy for measuring fine motor coordination in a controlled setting, it lacks key elements of real-world surgical environments. Specifically, it does not replicate the complexities of live surgery, such as dynamic tissue properties, intraoperative bleeding, anatomical variability, time pressure, and the psychological stress inherent to operating rooms. In addition, the VR system employed limited haptic fidelity, providing only binary (on/off) vibration cues, whereas real surgical systems often incorporate continuous, graded force feedback to convey subtle differences in tissue resistance and force application. These simplifications limit the validity of our findings and may attenuate the relevance of task performance to actual operative outcomes.

Finally, the observed sex imbalance between subgroups may also affect generalizability, given that visuomotor coordination and task performance can vary by sex. Future work should include more balanced samples, as well as explore how varying levels of visual and haptic fidelity can be optimized to match users’ skill levels and clinical experience. Understanding these interactions in greater depth will be essential for designing training systems and robotic interfaces that adapt appropriately to the needs of both novice learners and experienced surgeons, potentially enhancing surgical training and patient care.

### Implications and future directions

Our findings suggest important implications for the design of training programs, particularly in simulated environments involving delayed visual feedback. Since novices demonstrated the greatest benefit from real-time haptic cues under latency, we recommend that simulation-based curricula incorporate haptic-augmented training in the early stages of skill acquisition. As learners gain proficiency and develop more stable internal models, the reliance on haptic feedback can be progressively reduced. This graduated approach may foster the development of more adaptable visuomotor control strategies, ultimately enhancing performance in conditions with suboptimal or delayed sensory input.In addition, our findings have also clear implications for telesurgery system design. While minimizing visual latency remains critical, unavoidable delays, such as those in remote or low-bandwidth settings, require adaptive solutions. Systems could implement real-time sensory-integration algorithms that adjust the weighting of visual and haptic feedback based on operator performance and expertise. For example, haptic input may be upweighted for novices during high visual latency, while experienced users may benefit from sustained reliance on predictive visual strategies. Such adaptive feedback modulation could optimize performance across varying conditions and user profiles.

Future research should extend these findings to surgical residents and attendings using more ecologically valid setups, including realistic force feedback and tissue simulation. Investigating how experienced clinicians respond to delayed vision and varying haptic feedback will help to generalize the results to real-world settings. Additionally, studies should explore whether training under delayed visual feedback with variable haptic input can serve as a form of “pre-training” enhancing surgeons’ adaptability to latency and improving performance in teleoperative environments.

## Conclusion

In summary, 200 ms visual latencies increased error time by 2.8 s, whereas haptic delays up to 200 ms had no significant effect. Under delayed vision, novices use haptic feedback to stabilize performance, but experts appear to be disrupted by real‐time haptic cues that conflict with delayed vision. For real‐world telesurgery, maintaining visual feedback latency below 100 ms is critical; when not possible, haptic cues can mitigate the performance drop—but only for less experienced users.

## Supplementary Information

Below is the link to the electronic supplementary material.

Supplementary Material 1

## Data Availability

The generated and analyzed dataset is available from the corresponding author upon reasonable request. Owing to participant confidentiality and institutional regulations, raw data cannot be made publicly available but can be shared anonymously upon request for research purposes.

## Abbreviations

GVS: General visuomotor skills
LE: Laparoscopic experience
PPT: Purdue Pegboard Test
VR: Virtual reality
WLG: Wire loop game
FLS: Fundamentals of laparoscopic surgery
HTC VIVE Pro: HTC VIVE Pro Virtual Reality Kit
VF: Visual feedback
HF: Haptic feedback
VF0-HF0: Real-time visual and haptic feedback
VF0-HF-200: Real-time visual feedback and 200 ms delayed haptic feedback
VF0-noHF: Real-time visual feedback and no haptic feedback
VF200-HF0: 200 ms delayed visual feedback and real-time haptic feedback
VF200-HF200: 200 ms delayed visual and haptic feedback
VF200-noHF: 200 ms delayed visual feedback and no haptic feedback
