# Supplementary material for: Impact of laparoscopic training, haptic feedback and visual haptic latency on virtual fine motor performance
Source: Sci Rep. 2025 Sep 12;15:32480. doi: 10.1038/s41598-025-18862-6 (PMC12432154; doi:10.1038/s41598-025-18862-6)
Supplement: Supplementary file 1 — Supplementary Material 1 [file 41598_2025_18862_MOESM1_ESM.docx]

**Supplement**

**Supplementary Table 1:** Participants’ gaming experience of the complete cohort (n = 57).

| **item** | **number (n)** | **percent (%)** |
| --- | --- | --- |
| active gaming | 3 | 5.3 |
| prior gaming experience | 11 | 19.3 |
| absent gaming experience | 43 | 75.4 |
| complete cohort | 57 | 100 |


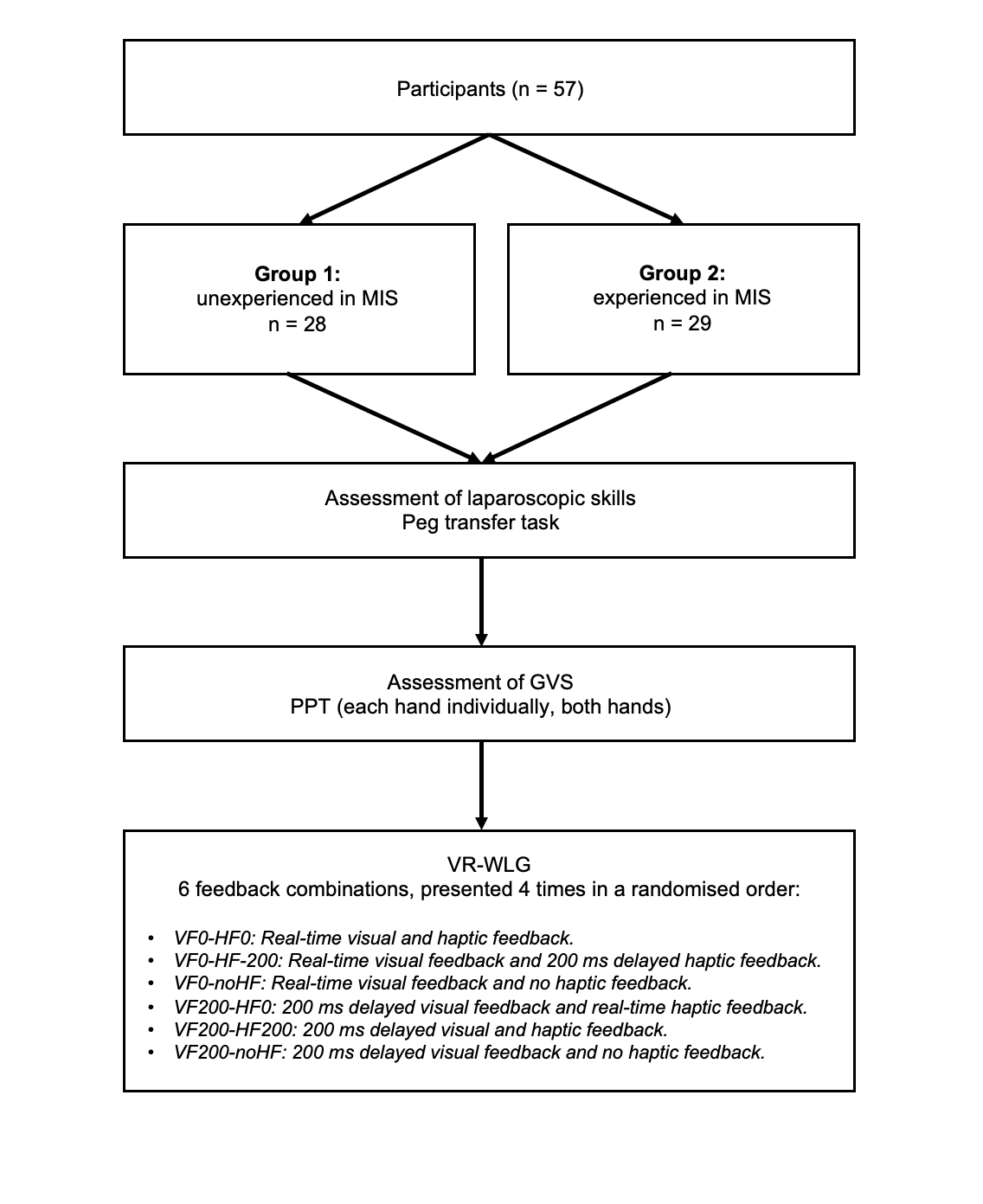


**Supplementary Fig. 1:** Trial scheme visualizing the study design.
